# Glucagon-like peptide-1 receptor agonists and type 1 diabetes: a potential game changer?

**DOI:** 10.3389/fendo.2024.1520313

**Published:** 2025-01-21

**Authors:** Ortal Resnick, Fernando Bril, Giovanna Beauchamp

**Affiliations:** ^1^ Division of Pediatric Endocrinology and Diabetes, University of Alabama at Birmingham, Birmingham, AL, United States; ^2^ Division of Endocrinology, Diabetes and Metabolism, University of Alabama at Birmingham, Birmingham, AL, United States; ^3^ UAB Comprehensive Diabetes Center, University of Alabama at Birmingham, Birmingham, AL, United States

**Keywords:** obesity, adiposity, insulin resistance, overweight, weight loss

## Abstract

This mini review explores the increasing prevalence of obesity in type 1 diabetes (T1D) and the challenges patients face in achieving optimal glycemic control with current treatments. It discusses the evidence supporting the use of glucagon-like peptide-1 receptor agonists (GLP-1RA) as potential adjunctive therapy in T1D to reduce weight and improve insulin resistance. Potential benefits need to be weighed against the risk of hypoglycemia and lack of long-term data.

## 1 Introduction

Since its introduction in 1921, insulin has changed the management and prognosis of patients with type 1 diabetes (T1D). New insulin formulations, together with advancements in insulin delivery and glucose monitoring technology, have changed the landscape for people with T1D. Notwithstanding these developments, only about 20% of patients with T1D achieve adequate glycemic control based on current targets (1). In addition, weight gain remains a significant concern for patients with T1D on intensive insulin therapy.

Based on recent data from National Health and Nutrition Examination Survey (NHANES) 2021-2023 (2), the prevalence of obesity in adults is 40.3% in the USA. Patients with T1D are equally affected by the obesity epidemic, and they have experienced a rapid increase in the prevalence of obesity in the last few years (3–6). Therefore, there is a strong need for new interventions to help manage obesity and hyperglycemia in T1D.

Glucagon-like peptide-1 receptor agonists (GLP-1RA) are effective to treat adult and pediatric populations with type 2 diabetes (T2D) and/or obesity, and they have an established safety profile. Their use in these populations has been associated with hemoglobin A1c reduction, significant weight loss, and a decrease in long-term microvascular and macrovascular complications (7–11). Even in patients without diabetes, semaglutide showed a decrease in cardiovascular events by ~20% (12). Recent American Diabetes Association (ADA) guidelines recommend GLP-1RA for weight management in T2D, and also GLP-1RA as first-line therapy in patients with T2D and established cardiovascular disease (13). However, their use in T1D is not recommended by any current guidelines. This likely responds to the fact that patients with T1D were excluded from large randomized, controlled trials (RCTs) assessing cardiovascular and renal outcomes with the use of these drugs (7, 8, 11). Moreover, evidence from RCTs in T1D has not consistently shown benefits in A1c reduction, insulin dose reduction, or other outcomes. However, most of these studies were done with liraglutide or other daily GLP-1RA, which are not as effective for weight loss as the newer GLP-1RA (e.g., semaglutide or tirzepatide). In addition, many of the studies were not even designed to target patients with elevated BMI, who are the subjects likely to benefit the most from these compounds. One could hypothesize that the weight loss benefit of these drugs can be, at least in part, extrapolated to patients with T1D as it is mainly driven by appetite suppression (14).

This mini-review discusses clinical studies evaluating adjuvant treatment with GLP-1RA in patients with T1D as an opportunity to improve glycemic control, achieve weight loss, and decrease total daily insulin (TDI) requirements in these patients.

## 2 Obesity in type 1 diabetes

Obesity has become a significant global health burden (15), and patients with T1D, who were historically characterized as lean, are nowadays found to be overweight or obese with increasing frequency in clinics. The prevalence of overweight and obesity has increased in T1D in pediatric and adult populations, and this has occurred at a faster pace than in the general population (3–5). In a recent study in the USA, overweight and obesity were reported in 34% and 28%, respectively, of patients with T1D (6). Currently, patients with T1D have a similar prevalence of overweight and obesity compared to the general population (2).

**Figure 1:**
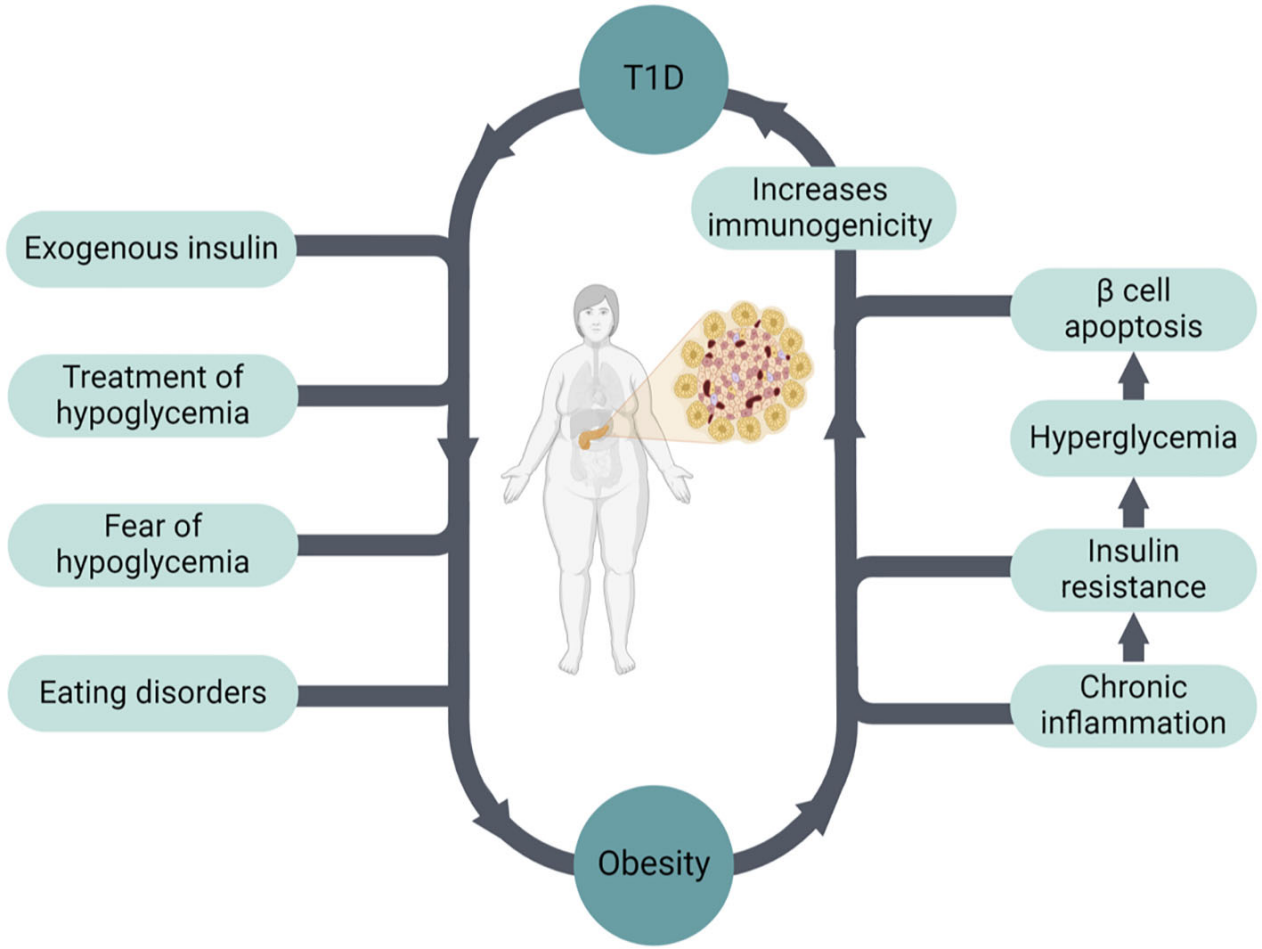
The bidirectional relationship between obesity and T1D.

Intensive insulin treatment improves glycemic control and reduces the risk of microvascular complications. However, it is considered an important risk factor for weight gain (16, 17) (**Figure 1**). In addition, prevention and treatment of hypoglycemia with excessive carbohydrates also contribute to weight gain. Fear of hypoglycemia can also lead to a reduction of physical activity and sedentarism, coupled with overeating. Other important contributing factors are eating disorders and depression, which are more common in patients with T1D compared to the overall population (18, 19). Weight gain causes insulin resistance (IR), which results in higher insulin requirements (20). Hyperinsulinemia is a key factor driving IR, thus leading to a positive feedback loop (i.e., hyperinsulinemia > IR > hyperinsulinemia).

Obesity increases the risk of obesity-related as well as diabetes-related complications in patients with T1D, including micro- and macrovascular complications, various types of cancer, and overall mortality (17, 20–22). For example, Wallace et al. showed that obesity in patients with T1D is associated with an increased risk of chronic kidney disease (CKD) compared with T2D (23). Obesity may also increase the risk of developing T1D (24, 25), and this could be one of the factors explaining the increasing incidence of T1D worldwide. Based on this hypothesis, obesity drives IR, which leads to hyperglycemia causing pancreatic β cell apoptosis. This process increases immunogenicity, which in turn leads to autoimmunity and the development of T1D in genetically predisposed patients (26, 27) (**Figure 1**).

## 3 Insulin resistance in type 1 diabetes

Insulin resistance is associated with micro- and macrovascular complications in patients with T1D (28). In these patients, IR is likely multifactorial and not fully understood (**Supplementary Figure 1**). While increased adiposity (i.e., overweight and obesity) is the usual driver of IR, other factors may play a role in the development of IR in T1D. For example, a family history of obesity and T2D may be an independent risk factor for obesity and IR in patients with T1D (29, 30). In a small study, Donge et al. compared IR measured by the gold-standard hyperinsulinemic euglycemic clamp in lean patients with T1D and healthy controls. They observed that patients with T1D were more insulin resistant than their BMI-matched counterparts at the levels of the liver, skeletal muscle, and adipose tissue (31). Similar results were found by larger studies (32). These findings suggest that there are additional factors contributing to IR other than obesity in T1D.

Insulin resistance in T1D could also be related to the insulin administration route. In subjects without type 1 diabetes, insulin is secreted from the pancreas into the portal vein, where 50-80% of insulin is metabolized in the first hepatic pass. On the contrary, exogenous insulin is absorbed from the subcutaneous tissue to the peripheral circulation, resulting in relative peripheral hyperinsulinemia and hepatic hypoinsulinemia (i.e., low portal-to-peripheral insulin ratio) (29). Indeed, peripheral insulin levels are ~2-fold higher in patients with T1D compared to patients matched for hyperglycemia (33). Compared to patients with MODY 2, who were well-matched for hyperglycemia and obesity, patients with T1D were significantly more insulin resistant, suggesting that IR is driven by peripheral hyperinsulinemia and not hyperglycemia (34). Indeed, peripheral insulin level was the strongest determinant of insulin resistance in this study. It is well-established that elevated peripheral insulin levels can modify insulin receptor expression and affect insulin sensitivity in skeletal muscle and adipose tissue (35). In support of this, when insulin is infused to healthy individuals without diabetes to levels observed in patients with T1D on insulin treatment, insulin sensitivity decreases (36). Moreover, when insulin administration route is changed from subcutaneous to intraperitoneal, glycemic control improves with lower insulin requirements, suggesting an improvement in insulin resistance (37). It has also been suggested that hepatic hypoinsulinemia may reduce insulin-like growth factor 1 (IGF-1) levels, which may contribute to IR as well by increasing growth hormone and IGF binding globulins (29).

Defining IR in patients with T1D in the clinical setting is also challenging. While we can assume that increasing insulin requirements would be a surrogate marker, this will depend on the carbohydrate intake of the patients. The hyperinsulinemic euglycemic clamp technique is the gold standard for measuring insulin sensitivity. However, this technique is impractical in the clinical setting and it is mostly used for research purposes. Other methods to measure IR, such as homeostatic model assessment for insulin resistance (HOMA-IR) and quantitative insulin sensitivity check index (QUICKI) are based on the measurement of fasting insulin and glucose. Therefore, these equations cannot be used in patients with exogenous insulin use (38). Estimated glucose disposal rate (eGDR) is a validated tool to estimate insulin sensitivity in type 1 diabetes using HbA1c, waist circumference, and presence of hypertension (39). However, its clinical usefulness remains uncertain.

Despite important knowledge gaps in our understanding of IR in T1D, targeting IR in overweight/obese patients with T1D may decrease the risk of diabetes complications, contribute to weight loss, and improve glycemic control.

## 4 Glucagon-like peptide-1 receptor agonists in type 1 diabetes

Glucagon-like peptide-1 (GLP-1) is secreted in response to food consumption from intestinal L-cells. GLP-1 is a multifaceted hormone, and the use of its analogs is associated with broad metabolic effects: they increase insulin secretion, decrease glucagon release, increase glucose uptake in muscles, decrease glucose production in the liver, improve lipid profile, slow gastric emptying, and increase satiety leading to weight loss (40). GLP-1 regulates inflammatory response by lowering the release of pro-inflammatory cytokines such as tumor necrosis factor-alpha (TNF-α), interleukin 6 and interleukin 1β, and stimulates activation of regulatory T cells. Specifically, GLP-1 reduces islet inflammation, inhibits apoptosis, and induces β cell proliferation in experimental models (41–43). This led to the consideration of GLP-1RA as medications that could change β cell function and survival in patients with T1D. Moreover, in patients with T2D, these compounds have shown cardiovascular, neurological, and renal protection (9, 12, 40, 44, 45), complications also frequently seen among patients with T1D.

GLP-1RA have been used to treat T2D for ~20 years, and they have transformed T2D management in pediatric and adult populations (9, 10, 44). One can hypothesize that their effects on glycemic control and reduction in long-term microvascular and macrovascular complications in patients with T2D might be equally beneficial to people with T1D, especially if overweight or obese. GLP-1RA can potentially improve quality of life through weight loss and reduction in insulin requirements. In addition, GLP-1RA reduce glucagon secretion, which can lessen postprandial hyperglycemia in patients with T1D (46, 47). However, there is concern about an increased risk of hypoglycemia and ketotic hyperglycemia. A recent study showed that, although not approved for T1D in the USA, GLP-1RA have been increasingly prescribed in patients with T1D (i.e., from 0.3% in 2010 to 6.6% in 2023) (48). As expected, the greatest increase in GLP-1RA prescriptions was among patients with T1D and obesity.

### 4.1 HbA1c reduction

#### 4.1.1 Uncontrolled studies

In a small, uncontrolled study, weekly semaglutide in 10 patients with newly diagnosed T1D, mean HbA1c improved dramatically from 11.7 ± 2.1% to 5.7 ± 0.4% at 12 months (49). However, results are difficult to interpret as this study was uncontrolled and fasting baseline C-peptide levels were relatively high at 0.65 ± 0.33 ng/mL. It is possible that some of those patients were in honeymoon period, or had an alternative diagnosis, such as ketosis-prone diabetes or latent autoimmune diabetes in adults (LADA). Due to the retrospective nature of the data, other confounding factors may have affected the results as well. In ‘real-world’ studies evaluating the efficacy and safety of GLP-1RA in patients with T1D, HbA1c had significant reductions of 0.4-0.5% (50, 51). Because these studies allow for concomitant insulin adjustments, and they lack a controlled group (i.e., placebo) interpretation of the A1c reduction is very difficult.

#### 4.1.2 RCTs

Several blinded, placebo-controlled RCTs have demonstrated a significant reduction in HbA1c when adding GLP-1RA to either multiple daily injections (MDI) or continuous subcutaneous infusion of insulin (CSII), ranging between 0.1-0.7% (52–61) (**Table 1**). The high heterogeneity of the results likely responds to different baseline A1c, variable rates of obesity, as well as different titration protocols to adjust insulin doses. Subgroup analysis in patients with positive baseline C-peptide levels showed a more robust reduction of HbA1c (i.e., 0.69-0.83%) on GLP-1RA in this group (56, 57). Overall, a meta-analysis of RCTs showed that A1c reductions were −0.28%, −0.21%, and −0.17% for liraglutide 1.8, 1.2, and 0.6 to 0.9 mg, respectively, and −0.17% for exenatide (62). Larger and longer studies are needed to evaluate this further.

**Table 1:** Summary of randomized, controlled trials assessing GLP-1RA in patients with T1D.

Study	Treatment		n	Duration (weeks)	Δ HbA1c (%)	Δ Body weight (%)	Δ TDI (%)	Hypo-glycemia	Primary endpoint	DM stage	Insulin dose titration with GLP-1RA initiation	BMI Inclusion criteria (kg/m^2^)
Frandsen et al., 2015 (59)	Liraglutide 1.2 mg		36	12	-0.1%	-5.7%	-7%	~	HbA1c	No β cell reserve	-25% bolus -10% basal	18-28
Lira-1 trial 2016 (58)	Liraglutide 1.8 mg		100	24	-0.2%	-7.3%	-15%	↓	HbA1c	> 1 year	-33% bolus -25% basal	>25
Kuhadiya et al., 2016 (60)	Liraglutide	1.2 mg	63	12	-0.48%	-4.9%	-14.3%	~	Weekly BG levels	> 1 year	-25% bolus -10% basal Only if HbA1c<7.5%	none
		1.8 mg			-0.12%	-5.4%	-16.8%	~				
ADJUNCT ONE trial 2016 (57)	Liraglutide	1.2 mg	1,398	52	-0.15%	-4.2%	-2%	↑	HbA1c, TDI, and weight	> 1 year	-25% TDI, plus -10% with dose escalation	≥20
		1.8 mg			-0.20%	-5.7%	-5%	↑				
ADJUNCT TWO trial 2016 (56)	Liraglutide	1.2 mg	835	26	-0.23%	-4.5%	-7%	↑	HbA1c	> 1 year	-25% TDI, plus -10% with dose escalation	≥20
		1.8 mg			-0.35%	-5.8%	-10%	~				
Lira Pump trial 2020 (55)	Liraglutide 1.8 mg		44	26	-0.7%	-7.4%	-15%	~	HbA1c	> 1 year	-15% bolus -10% basal	>25
MAG1C trial 2020 (54)	Exenatide 10μg TID		105	26	-0.1%	-5.0%	-15%	~	HbA1c	> 1 year	Adjusted individually	>22
Pozzilli et al., 2020 (53)	Albiglutide 50 mg		61	52	+0.1%	+0.2%	NR	~	Stimulated C-peptide	New *	Algorithm based on glucose levels	<32
DIAMOND-GLP-1 trial 2023 (52)	Dulaglutide 1.5 mg		18	24	-0.1%	-6.4%	NR	↓	HbA1c	With β cell reserve^#^	Not specified	16-30
NewLira trial 2024 (61)	Liraglutide 1.8 mg		68	52	-0.37%	-4.4%	-70%	↓	Stimulated C-peptide	New ^	-25% bolus -10% basal	>20

*4-8 weeks since insulin initiation.

^#^C-peptide levels above 15 pmol/l.

^up to 6 weeks from diagnosis.

All studies were blinded and placebo-controlled. Changes reflect the placebo-subtracted effect. n, number of subjects; TDI, total daily insulin; DM, diabetes; ↑, significantly elevated compared to placebo; ~, unchanged compared to placebo; ↓, decreased compared to placebo.

### 4.2 Total daily insulin and C-peptide secretion

#### 4.2.1 Uncontrolled studies

Using weekly semaglutide, prandial insulin was discontinued in all patients, and basal insulin was discontinued in 7 out of 10 patients. In addition, increased C-peptide levels and better glycemic control during the year of observation were noted (49). However, as aforementioned, due to the lack of a placebo group, high baseline C-peptide levels, and concomitant use of a restricted carbohydrate diet, these results are difficult to interpret or extrapolate to other populations. A small, short study had two patients on liraglutide with positive postprandial C-peptide at baseline completely discontinued insulin treatment with good glycemic control (63). In this study, insulin dose reduction was higher in patients who had a positive C-peptide at baseline, emphasizing that early initiation may be of benefit. Lower insulin requirements are also noted in ‘real-world’ studies (50, 51).

#### 4.2.2 RCTs

In RCTs, insulin dose reductions were observed with the addition of GLP-1RA (52–61) (**Table 1**). Only dulaglutide treatment did not decrease TDI, however this was a small study (n=18) (52). Although β cell function improves in T2D patients, treatment with liraglutide in T1D did not significantly change mean C-peptide concentration (56–58). Similar results were seen in the study by Pozzilli et al., where the efficacy of once weekly albiglutide on preserving β cells was assessed in patients with newly diagnosed T1D. C-peptide levels were not significantly different between the intervention and placebo groups (53). However, all studies were performed on stage 3 type 1 diabetes, so whether initiation of these drugs at earlier stages of the disease can help prevent β cell function loss is unknown.

### 4.3 Weight change

#### 4.3.1 Uncontrolled studies

While insulin monotherapy causes consistent weight gain, adding GLP-1RA leads to consistent weight loss. Weight loss appears to be more rapid in the first 10-12 weeks, but it continues to decrease at a slower rate thereafter (64). In ‘real-world’ studies weight loss was significant after 12 months and 3 years of treatment (50, 51).

#### 4.3.2 RCTs

In RCTs (**Table 1**) (52–61), weight loss was observed in all trials, except with albiglutide. Weight loss was similar in patients on CSII, with a mean weight loss of 6.3 kg (55). Weight loss was also similar in studies with dulaglutide and exenatide (52, 54). Of note, not all studies focused on overweight or obese patients, with many allowing patients with BMI ≥20 kg/m^2^. Even in these studies including patients without overweight or obesity, significant weight loss was observed. As expected, those studies limiting BMI to >25 kg/m^2^ had a larger reduction in weight. In a meta-analysis with patients with T1D, weight loss with liraglutide 1.8 mg was estimated at ~5 kg compared to placebo (62), and there was a dose-response effect.

### 4.4 Safety

Hypoglycemia is common in patients with T1D. The average patient with T1D experiences two episodes of symptomatic hypoglycemia a week (65). In a survey of 436 participants with T1D, 72% of those who drive a vehicle reported having hypoglycemia events while driving, and 4.3% reported having a vehicular accident due to hypoglycemia in the previous 2 years (66).

In RCTs comparing GLP-1RA and placebo, insulin doses were reduced before GLP-1RA initiation to avoid hypoglycemia, and close monitoring and insulin adjustments were done periodically. As can be observed in **Table 1**, basal insulin was reduced by ~10-25% and bolus doses by ~15-33% in RCTs. Therefore, hypoglycemia rates may not reflect hypoglycemia rates observed in clinical practice. However, even in large ‘real world’ cohorts of patients with T1D on GLP-1RA, low rates of hypoglycemia were observed (50, 51).

ADJUNCT ONE and TWO trials, the 2 largest studies with GLP-1RA in T1D, reported increased rates of hypoglycemia. The ADJUNCT ONE trial found that symptomatic hypoglycemia had a dose-related effect with a rate ratio between 1.27 and 1.31 on doses of 1.2 and 1.8 mg of liraglutide, respectively. While the ADJUNCT TWO also reported an increased rate ratio of symptomatic hypoglycemia of 1.33 on 1.2 mg liraglutide, events were not significantly different with the 1.8 mg dose in that study. In other studies, GLP-1RA addition did not increase the risk of hypoglycemia. Overall, results are heterogeneous, but they point towards a small increase in the risk of hypoglycemia. However, adequate adjustment of insulin doses and close monitoring after GLP-1RA initiation may decrease this risk. Of note, a meta-analysis by Park et al. showed no differences in the frequency of symptomatic hypoglycemic events or severe hypoglycemia (62).

As insulin doses are reduced to prevent hypoglycemia, there is also a potential risk of hyperglycemia and eliciting diabetic ketoacidosis. The risk of ketotic hyperglycemia did not increase in the aforementioned meta-analysis (56). Among RCTs, increased ketotic hyperglycemia events were reported in the ADJUNCT trials in a dose-related manner (48, 49). However, it was not reported in smaller trials (53–55, 58–61), and the DIAMOND-GLP-1 trial reported a decrease in the frequency of events (52, 52).

In retrospective studies, acute pancreatitis was not reported in patients with T1D treated with GLP-1RA (45, 50, 51). In 2014, the FDA and the EMA found no causal association between GLP-1RA and pancreatic adverse events (67). Pancreatitis was not reported in the RCTs (52–61).

While some of the trials reported a decrease in appetite as a side effect, like the ADJUNCT trials (53–58, 60), it should be noted that this is one of the main mechanisms of action of these drugs to achieve weight loss. Nausea and gastrointestinal adverse effects were reported in about half of patients with T1D on liraglutide (56–58). However, a relatively low discontinuation rate was reported, 0-15%. For example, in the ADJUNCT ONE trial, the rate of nausea with the highest dose of liraglutide (1.8mg) was 49.6% compared to 12.1% in the placebo. Use of liraglutide 3mg daily in patients with obesity without diabetes was associated with nausea in 40.2% compared to 14.7% in the placebo (68). While it is difficult to compare these studies head-to-head, overall, it seems that gastrointestinal symptoms are relatively similar in patients with *vs*. without T1D. In a meta-analysis of GLP-1RA use in T1D, all adverse events were significantly higher in the GLP-1RA group, but there was no difference in serious adverse events (62).

### 4.5 Pediatrics

Only few small studies have assessed the use of GLP-1RA as adjuvant therapy in pediatric patients with T1D. A small trial including 8 patients with T1D showed improved postprandial hyperglycemia despite 20% insulin dose reduction (69).

## 5 Cardiovascular and renal outcomes with GLP-1RA in T1D

In patients with T2D, large RCT studies have shown that GLP-1RA treatment reduces the risk of cardiovascular and renal outcomes (7, 8, 11, 70). Moreover, the SELECT study showed that semaglutide significantly reduced the incidence of death from cardiovascular causes, nonfatal myocardial infarction, or nonfatal stroke (hazard ratio 0.80) (12). Unfortunately, due to different FDA regulations and safety concerns, it would be hard for this type of RCTs to concomitantly include patients with T1D and T2D, and therefore patients with T1D were excluded from these trials. Therefore, results cannot be extrapolated to the T1D population. However, observational data has shown that obesity in T1D is associated with higher risk of microvascular and macrovascular complications (17, 20–22). Therefore, one can argue that targeting obesity in T1D may share some of the benefits observed in T2D or in obesity without diabetes.

## 6 Practical issues when prescribing GLP-1RA in T1D

GLP-1RA are not currently approved for treatment of T1D, but they may still be covered by insurance if prescribed to treat obesity in these patients (although insurance coverage for obesity remains limited). Based on data from RCTs in T1D and those with patients with T2D, initiation of a GLP-1RA should be followed by a decrease of total insulin dose to reduce events of hypoglycemia (71). The amount of reduction of insulin dose should be determined on an individual basis based on prior diabetes control, risk of hypoglycemia and ketosis, type of GLP-1RA started, among others. In RCT studies on T1D, at the initiation of GLP-1RA therapy, bolus insulin was reduced ~25-33% and basal insulin ~10-25% (56–61) as observed in **Table 1**. In some of the studies, an additional 10% dose reduction was done with drug escalation. In the study by Kuhadiya et al. insulin dose at GLP-1RA initiation was only decreased if HbA1c was below 7.5% (60). In the Lira Pump study, they decreased basal insulin by 10%, but increased bolus by 15% (55). Only in the ADJUNCT trials an increase in ketotic hyperglycemia events were reported with these dose adjustments (56, 57).

Our current practice is to consider a ~20% bolus insulin decrease and a ~10% basal insulin decrease before GLP-1RA initiation, although prior A1c, risk of hypoglycemia, potential risk of ketosis due to reduced insulin doses, as well as other patient-specific factors should guide the final insulin dose changes. Further adjustments may be needed as patients experience weight loss. Rapid weight loss or prolonged fasting periods due to appetite suppression is likely to be associated with a higher risk of hypoglycemia, and therefore, should be avoided in patients with T1D. Titration of GLP-1RA based on the individual’s response in weight and glycemic control is recommended. Slow titration will also help decrease gastrointestinal symptoms that may arise. These symptoms improve with continuous use, as only 3% and 9% of patients reported nausea with the long- or short-acting GLP-1RA after 6 months of use, respectively (72). These medications, except exenatide, are safe for patients with mild to severe renal impairment without dose adjustments (73, 74). GLP-1RA can be used in patients with hepatic impairment, without dose adjustments, although they have not been extensively studied in these circumstances (71).

## 7 Conclusions

Obesity increases the risk of microvascular and macrovascular complications, various types of cancer, and overall mortality in patients with T1D. New approaches are needed to address the rising rates of overweight and obesity in the T1D population. Despite new insulin formulations and improvements in the technology of insulin pumps and continuous glucose monitoring, glycemic control continues to be suboptimal in T1D, with about 80% of patients not reaching the recommended goals. Therefore, GLP-1RA is a promising group of medications to treat obesity, improve glycemic control, and decrease the risk of complications in these patients.

Overall, studies have shown that these medications have an established safety profile in T1D, with only a modest effect on HbA1c, but significant weight loss, and a reduction in TDI. These effects occur at the expense of a slight increase in hypoglycemia, but careful titration of insulin doses may mitigate this risk. Weight loss compared to placebo was ~5% in RCTs. However, there is lack of RCTs in T1D using the newest generation of GLP-1RA or dual GLP-1/GIP agonists, which are associated with significantly more weight loss. Moreover, because patients with T1D were excluded from studies looking at cardiovascular and renal outcomes with the use of GLP-1RA, it remains unknown whether those benefits observed in patients with T2D or obesity without T2D translate to the T1D population. In addition, whether early initiation of GLP-1RA in patients with newly diagnosed T1D can preserve β cell function remains to be determined in large RCTs. Until more research is available, the use of these drugs in T1D should be done carefully, with a thorough discussion with patients about potential risks and benefits of this approach.
