# Supplementary figures and images for: Glucagon-like peptide-1 receptor agonists and type 1 diabetes: a potential game changer?
Source: Front Endocrinol (Lausanne). 2025 Jan 21;15:1520313. doi: 10.3389/fendo.2024.1520313 (PMC11790463; doi:10.3389/fendo.2024.1520313)

**Supplemental Figure 1.** Mechanisms leading to insulin resistance in patients with T1D.


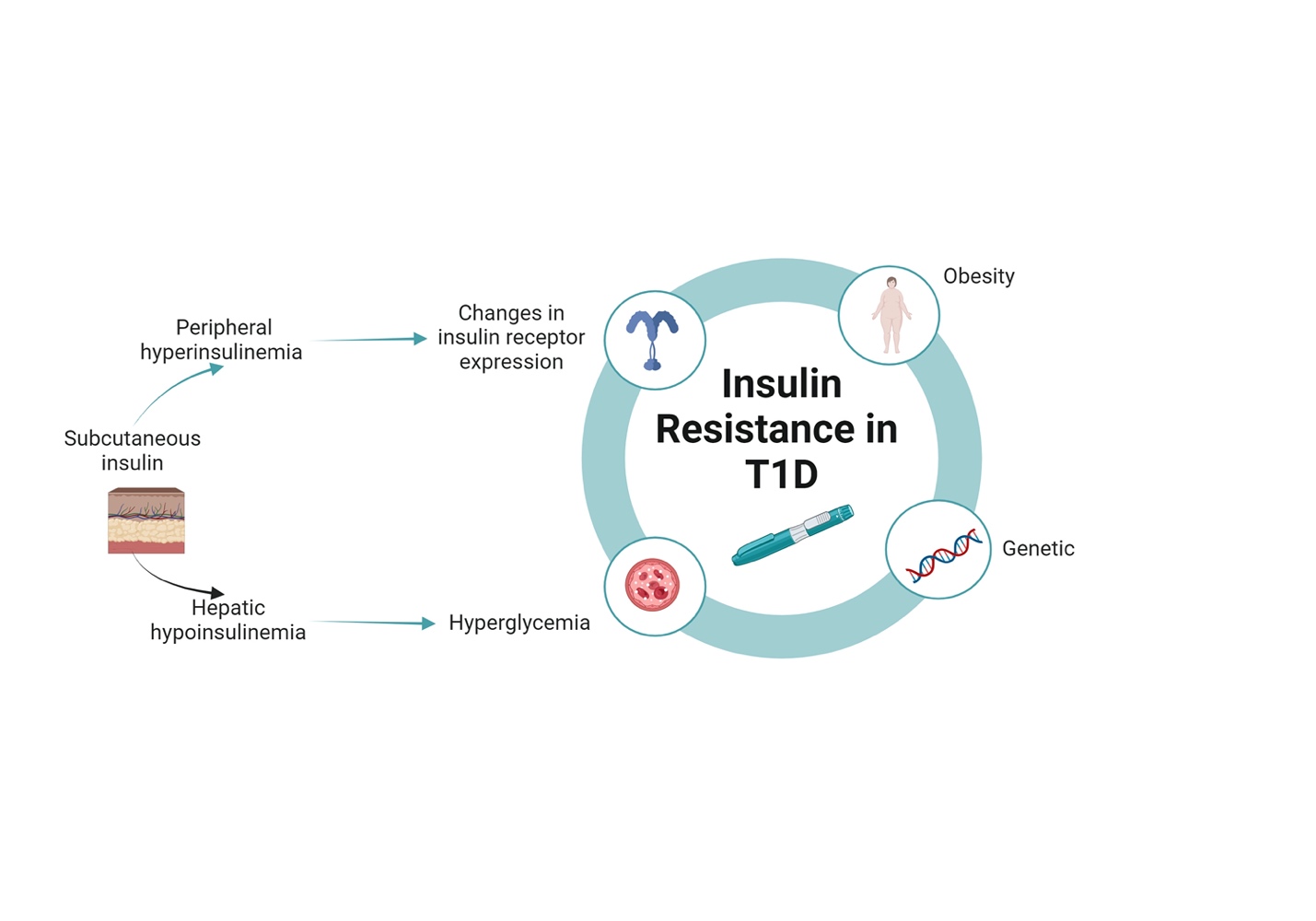

Supplement: Supplementary file 1 [file DataSheet1.docx]
